# Seeing the Unseen—Bioturbation in 4D: Tracing Bioirrigation in Marine Sediment Using Positron Emission Tomography and Computed Tomography

**DOI:** 10.1371/journal.pone.0122201

**Published:** 2015-04-02

**Authors:** Matthieu Delefosse, Erik Kristensen, Diane Crunelle, Poul Erik Braad, Johan Hygum Dam, Helge Thisgaard, Anders Thomassen, Poul Flemming Høilund-Carlsen

**Affiliations:** 1 Department of Biology, University of Southern Denmark, Odense, Denmark; 2 Department of Nuclear medicine, Odense University Hospital, Odense, Denmark; 3 Institute of Clinical Research, University of Southern Denmark, Odense, Denmark; University of Manchester, UNITED KINGDOM

## Abstract

Understanding spatial and temporal patterns of bioirrigation induced by benthic fauna ventilation is critical given its significance on benthic nutrient exchange and biogeochemistry in coastal ecosystems. The quantification of this process challenges marine scientists because faunal activities and behaviors are concealed in an opaque sediment matrix. Here, we use a hybrid medical imaging technique, positron emission tomography and computed tomography (PET/CT) to provide a qualitative visual and fully quantitative description of bioirrigation in 4D (space and time). As a study case, we present images of porewater advection induced by the well-studied lugworm *(Arenicola marina)*. Our results show that PET/CT allows more comprehensive studies on ventilation and bioirrigation than possible using techniques traditionally applied in marine ecology. We provide a dynamic three-dimensional description of bioirrigation by the lugworm at very high temporal and spatial resolution. Results obtained with the PET/CT are in agreement with literature data on lugworm ventilation and bioirrigation. Major advantages of PET/CT over methods commonly used are its non-invasive and non-destructive approach and its capacity to provide information that otherwise would require multiple methods. Furthermore, PET/CT scan is versatile as it can be used for a variety of benthic macrofauna species and sediment types and it provides information on burrow morphology or animal behavior. The lack of accessibility to the expensive equipment is its major drawback which can only be overcome through collaboration among several institutions.

## Introduction

Bioturbation refers to the physical effects of animals on their substratum [1]. It typically takes two forms in marine ecosystems: particle reworking and ventilation [1]. These two processes, which primarily result from the activities of benthic (usually burrowing) macroinvertebrates, may have significant ecological implications [1–4]. While sediment mixing by particle reworking occurs through faunal feeding, defecation and burrowing activities, gallery flushing with water by ventilation is primarily conducted for respiration and feeding purposes [1,5]. Ventilation has ecological importance both below and above the sediment surface [3,5]. This is particularly true when it occurs in blind-ended burrows because water pumped by the animal is forced to percolate through the porous sediment around the burrow. This advective bioirrigation and its oxidative transport have important consequences for biogeochemical cycles [3,6].

Descriptive bioturbation studies are challenging because they attempt to describe biologically driven dynamic 3D processes that occur in an opaque sediment matrix. Although various techniques have been developed for this purpose, no single experimental approach has yet provided dynamic 3D images of bioturbation [7,8]. Facing the lack of experimental options, the approach is currently to extract and combine the results obtained from a selected number of existing methods depending on the question addressed [5,9]. Although this approach has been used with success, it has obvious limitations. To date the most complete spatial and temporal description of sediment bioirrigation has been achieved using dynamic 3D models but these cannot stand alone as they lack proper experimental validation [8].

We present the hybrid medical imaging technique, positron emission tomography/computed tomography (PET/CT) to simultaneously measure ventilation and visualize biologically driven porewater advection in 4D. PET/CT is a radiologic procedure that merges two imaging techniques: (CT) computed tomography with transmission of x-rays and (PET) positron emission tomography with emission of gamma rays [10]. These techniques have separately proven useful in marine science, but they have never been combined. While CT scan provides 3D structural information of biogenic structures [11], the PET component offers 3D information of porewater flow dynamics [12]. A major advantage of these techniques is their non-destructive and non-invasive nature.

**Fig 1:**
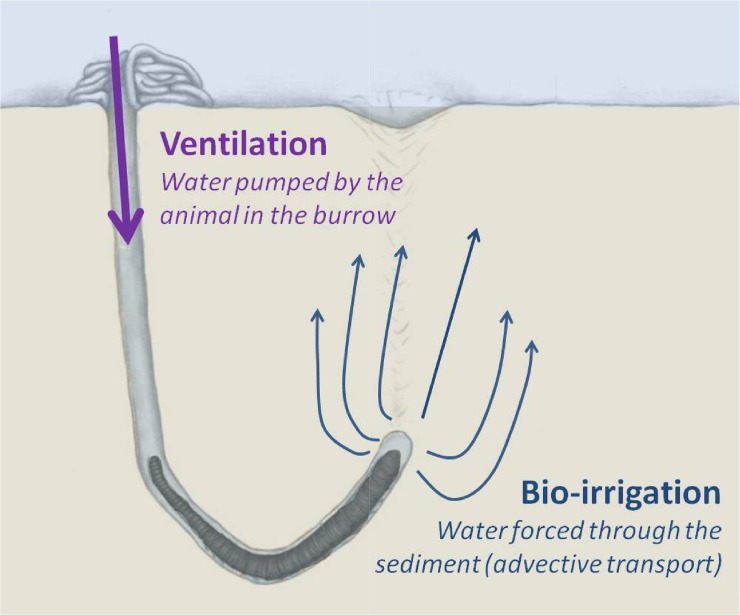
Schematic representation of ventilation and bio-irrigation in the blind-ended burrow of the lugworm, *Arenicola marina*. Lugworms ventilate intermittently oxic overlying water into their burrows to survive anoxic conditions. The flow of water passes over the gills of the worm and is subsequently forced (or bioirrigated) into the surrounding sediment. Porewater advection changes the sediment biogeochemistry dramatically. The extent and the scale of this effect depend on the ventilation activity of the animal and the distribution pattern of the irrigated water.

To prove the applicability of PET/CT, we obtained a dynamic 3D visualization of porewater advection induced by the burrowing polychaete *Arenicola marina* (or lugworm). *A*. *marina* typically lives in deep (>15 cm) blind-ended burrows, which are ventilated at a rate of about 1 ml water/min ([13]; Fig 1). The pumping activity and the associated bioirrigation generated by this species have been extensively studied because of its omnipresence in European coastal waters [14]; making it a perfect study object for a comparative methodological study.

### Basic principles of PET/CT

**Fig 2:**
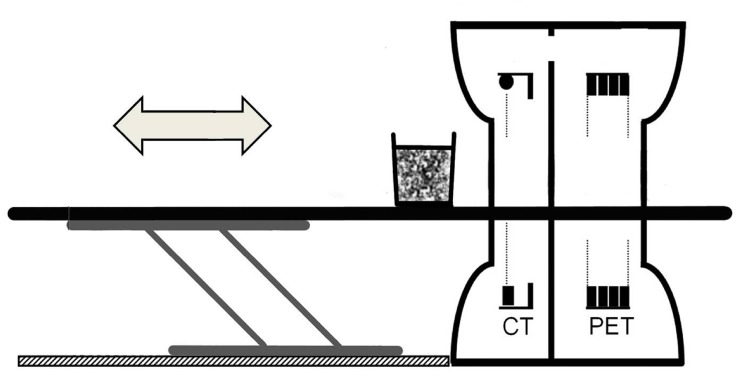
Schematic drawing of PET/CT scanner. The bucket containing sediment and lugworms is positioned on a table that can be moved accurately from the CT to the PET compartment of the scanner (Modified from [15]).

A PET/CT scanner combines two medical imaging techniques (Fig 2): computed tomography (CT) and positron emission tomography (PET). CT transmits x-rays through the studied object, whereas PET detects photons generated when positrons emitted by a radionuclide tracer inside the object is annihilated with electrons. Both techniques are tomographic *i*.*e*. the scanner generates a series of 2D cross-sections (thin slices), that are superposed by a computer algorithm to create a full 3D image of the object. Series of 3D images are acquired repeatedly over time to create a dynamic 3D image *i*.*e*. 4D. The CT scanner provides morphological information of the studied object, whereas the PET scanner yields information on the accumulation or transport of a radioactive tracer inside the object. Noticeably, the tracer is only used in very small quantities enabling the study of objects under physiologically normal conditions. PET/CT was commercialized in 2001 [15], and has been increasingly used in various fields of medicine, but especially in oncology for detection, staging and response evaluation of a wide array of cancers. The following descriptions introduce the general terminology and basic principles of CT and PET.

### Computed tomography (CT)

CT (computed tomography) is based on X-ray radiography and uses the X-ray attenuation properties of an object depending on its bulk density and its effective atomic number [16]. Thus, each voxel (i.e. 3D pixel) on the resulting X-ray image has a distinct contrast corresponding to the average specific attenuation of the voxel through the object. When the X-ray tube rotates in a spiral around the studied object, it allows the reconstruction of an image with good contrast at sub-millimeter resolution for each slice of the object that is read in Hounsfield units (HU).

### Positron emission tomography (PET)

Once the CT has been completed, the object is moved slowly and accurately by a motorized table to the PET where a radioactive tracer is (or has been) administered (Fig 2). Tracers may be a single radionuclide- for example fluoride ^18^F or an element that is incorporated into virtually any molecule—for instance H_2_^15^O. One key feature of the radioactive isotopes is that the chemical characteristics of the element are not altered no matter which isotope is applied. The radionuclides used in PET must be positron (β^+^) emitting isotopes. The most frequently used radionuclide in medical imaging is fluoride ^18^F, but oxygen ^15^O, nitrogen ^13^N, and carbon ^11^C are also commonly used [17]. The half-life of most radioisotopes used in medicine ranges from minutes ^15^O (2 min) to hours ^18^F (110 min) [17,18].

The principle of PET is based on the detection of two collinear photons (γ-rays) generated when the positron emitted by the radionuclide is annihilated with an electron [10]. The two opposing photons from each annihilation events are detected by a series of cylindrical detectors that surrounds the object (Fig 2). Then, each photon pair is assigned a set of spatial coordinates, from which 3D images can be generated by tomographic reconstruction. The transaxial field of view (FOV) is smaller than the detector ring diameter, and restricts the window over which images can be reconstructed for a given position. Image acquisition may be in *dynamic* or *static* mode depending to the desired outcome. A dynamic acquisition is a series of static images confined to a single position acquired over time with a temporal resolution of a few seconds. However, spatially displaced successive static scans can cover larger objects. For example, static scanning of a human body usually requires 20 to 30 min [12]. A benefit of PET is its quantitative capacity, which means that the amount of tracer can be measured exactly in any region of the object after correction for attenuation and photon scatter. Corrections are based on the density of the material as derived from the CT scan acquisition. On the other hand, inaccuracy may occur in the localization of the tracer because the scanner detects the opposing photons from the annihilation and not the decay itself. However, this inaccuracy remains typically less than a few mm, and depends on the energy of the positron and the density of the medium in which the positron travels [17].

## Materials and Methods

### Sample collection and preparation

Lugworms (*Arenicola marina*) and sediment were collected in spring 2011 from Bregnør Bight on the southeastern coast of Odense Fjord on the island of Fyn, Denmark (N: 55.4790 E: 10.6129; [19]). The sediment was passed through a 1 mm sieve to remove animals, gravel and shell debris before it was directly transferred to 10 plastic buckets (diameter: 25 cm). The buckets were filled with 9 liters of sediment to a height of 20 cm and covered by 1.5–2 liters of aerated seawater. The buckets were stored in 200 liter laboratory tanks containing well-aerated seawater at 21 ± 2°C and at a salinity of 20 ± 3 under a 12h/12h day/night cycle. The sediment was left to compact for 8–10 days until scanning. Lugworms (6 to 8 cm long) were collected from the same site 3 days before scanning. Intact animals were placed in Petri dishes containing aerated seawater to void their guts for at least 12 h before being weighed after brief blotting on a dry tissue. Worms were introduced to the buckets after careful transport to Odense University Hospital and left to establish burrows for 1 to 2 days before scanning.

### Scanning configuration and setup

All scans were acquired from a GE Discovery RX PET/CT scanner (General Electric Healthcare, Waukesha, WI, USA). A helical CT-scan was acquired using a standard CT protocol with a scan field of view of 70 cm. Data was reconstructed with a standard filter into transaxial slices with a field of view of 50 cm, matrix size of 512x512 (pixel size 0.98 mm) and a slice thickness of 3.75 mm. CT-scan was followed immediately by a dynamic PET-scan with field view of 70 cm. The CT-scan was used for attenuation correction and the scatter was modelled using single scatter simulation algorithm. The PET data was reconstructed into transaxial slices with a matrix size of 128x128 (pixel size 5.47 mm) and a slice thickness of 3.75 mm using iterative 3D OS-EM (2 iterations, 21 subsets) [20].

Five buckets with active worms were selected for scanning. The activity and condition of the lugworms were assessed by two visual criterions: (1) formation of fecal casts at the sediment surface confirmed that lugworms were alive and feeding and (2) formation of white *Beggiatoa*-like bacterial mats at the sediment water interface confirmed that lugworm irrigation moved sulfide-rich water upwards in the sediment [21].

One single CT scan was taken of each bucket for the structural description, whereas PET conditions were registered dynamically with an acquisition time of 15 seconds per frame. It is assumed that there was little change in the burrow geometry over the total time of the experiment (1 h) and that the slow and smooth movement of buckets on the sliding table during CT scan only had slight influence on worm position and activity. We conducted image selection based on the geometry of the burrow obtained from the CT scans. It was important for the experiment that burrows were contained within the FOV of the PET scanner (here, 15 cm in width) to obtain a dynamic acquisition.

Approximately 80 MBq of Na^18^F dissolved in 1 ml water were introduced directly to the continuously aerated overlying seawater. This corresponded to an initial concentration of less than 0.40 nM Fluoride. The mixture of the tracer and the overlying water was done by continuous aeration. Fluoride ^18^F was chosen because it had the longest half-life of the radionuclides readily available, and has been shown to be applicable in similar studies [12]. Fluoride is known for its strong affinity to CaCO_3_, Fe and clay (R.C. Aller, pers. comm.) suggesting that its use as a conservative tracer in sediments containing high levels of these elements should be avoided. The Bregnør Bight sediment consisted of quartz sand and contained only little carbonate (3.5% DW), iron (0.3 mmol.gDW^-1^) and clay (<5% DW) (S1 File). However, the extent of fluoride precipitation on the obtained results is unknown, but assumed to be relatively small [12]. The toxicity of ^18^F to lugworms was limited since it was introduced at very low levels and all lugworms were alive and in good condition at the end of experiment. Similarly, Roskosch et al. [12] exposed the larvae of *Chironomus plumosus* to the same fluoride levels and did not report any toxicity. Finally, the 110 min half-life of ^18^F seems to be optimal for determination of lugworm activity as it allows a recording time of several hours. Once the scanning was finished, the buckets were closed and the ^18^F was left to decay for more than 48 hours before the sediment characteristics were determined.

### Sediment characterization

Sediment characteristics were measured on two cores obtained simultaneously by introducing two core liners (i.d. 5 cm) into the buckets. The cores were sliced at 1 cm intervals to 20 cm depth and the following measurements were done for each layer. Density (g·cm^-3^) was measured as the weight of a known volume of sediment; water content (%) as the weight loss after drying for 24 h at 105°C; and organic content as the weight loss after combustion for 5 h at 520°C [22]. Porosity was calculated from of water content and density. Grain size was measured by passing about 20 g of wet sediment through a Wentworth series of sieves of decreasing size. The median grain size and sediment type were calculated using Gradistat 4.0 [23].

### CT and PET data extraction

#### Burrow dimensions from computed tomography

Visualization of CT images was done using Advantage Workstation v. 4.4. (General Electric Healthcare, Waukesha, WI, USA). The 3D images of the water-filled burrow were created as the difference between attenuation of seawater and sand [24]. The burrow dimensions were quantified from 10–30 sections for which both diameter and length were measured to calculate the average diameter and the total length of the burrow. More accurate measurements can be obtained, but it requires a more tedious procedure [25,26].

### 4D visualization of bio-irrigation

The ^18^F was rapidly mixed homogeneously in the overlying water after addition. The initial images from PET obtained 1–2 min after tracer addition should therefore provide a valid starting point for a 4D visualization of water transport. Subsequent 3D images were then acquired dynamically with a temporal resolution of 1 minute and used as time lapse frame to create a dynamic 3D representation of the bioirrigation pattern. We could achieve much higher frequency, but the chosen time interval was sufficiently to visualize the ventilation pattern of the lugworm in a sensitive manner. A higher temporal resolution would result in more noisy images. The 3D PET images and CT images were combined on Advantage Workstation v. 4.4. (General Electric Healthcare, Waukesha, WI, USA).

### Quantification of ventilation and bioirrigation

Corrections for e.g. tracer attenuation were done automatically when PET and CT images were combined. However, it was unknown whether the correction method used in clinical studies could be transferred to measurements in sediment. The radioactivity measurements were therefore validated by placing 6 closed spheres with a known concentration of Na^18^F (4 kBq/ml) in the water above the sediment and inside the sediment (volumes ranging between 0.5 ml and 27 ml). Based on the expected radioactivity in the various spheres, it was confirmed that PET quantification of radioactivity in the sediment was valid within an accuracy of ca. 5%.

**Fig 3:**
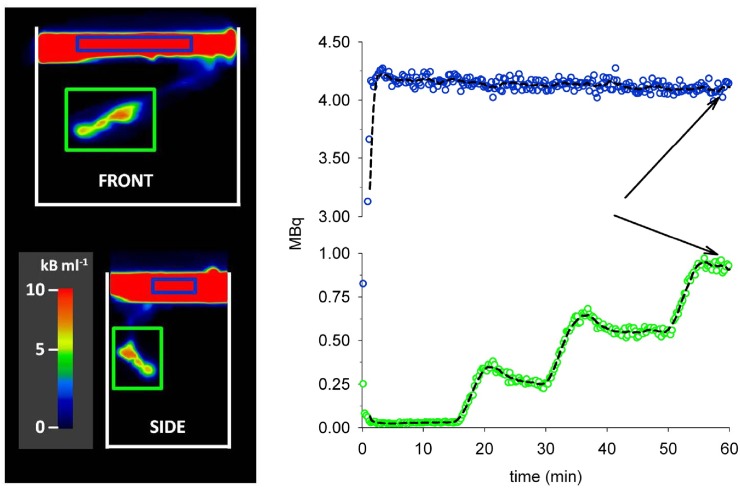
3D visualization and quantification of water movement between overlying water and the sediment compartments. Left pictures: The location of radioactivity within the reference region of interest (ROI_ref_) is framed in blue and the sediment ROI (ROI_sed_) is framed in green on a 3D picture (front and side view) obtained from the PET scanner after 59 minutes. The temporal change in the radioactivity within each ROI is shown on the scatter plots (colors of symbols correspond to the colors of frames). Arrows indicate the timing of pictures to the left. Front (25 cm) x Side (15 cm).

The volume of water transported by the lugworm into the sediment was estimated from the accumulation of ^18^F activity in the sediment. Two 3D regions of interest (ROI) were identified from the PET images and the total radioactivity in each of these ROI was recorded for each time frame (i.e. every 15 seconds). The reference ROI (ROI_ref_) was placed in the overlying water to determine radioactivity of the source water pumped by the lugworm into the sediment. Sediment ROI (ROI_sed_) was defined to encompass the volume of sediment affected by the bioirrigated zone as seen from the PET images (Fig 3).

The volume of water pumped into the sediment was calculated as (*V* = *A_sediment* / *c*), where *A_sediment* is the accumulated ^18^F radioactivity (MBq) in the sediment ROI and c is the radioactivity (MBq/ml) in the overlying water. The underlying assumptions are that the background radioactivity of the water contained inside burrows is negligible and that the radioactivity in the overlying water remains constant (after decay correction) during the experiment. The latter assumption was supported by the low lugworm pumping (<1 ml/min), which corresponded to less than 5% of the overlying water during the 1 hour experiment. This assumption was further verified a posteriori (Fig 3). To determine the volume of sediment affected by bioirrigation, the volume of water injected by the worm was divided by the sediment porosity. Finally, the porewater flow rate was computed by dividing the pumping rate of the lugworm by the cross section of the burrow calculated from the CT images.

## Results

### Sediment characteristics

The sediment consisted of organic poor (<1%) moderately well sorted fine sand with median grain size of about 0.22 ± 0.02 mm. The average density of the sediment was about 1.78 g·cm^-3^ and the water content was 16–20%. The average porosity of the sediment was constant below 1 cm at a level of about 0.27.

### Burrow dimensions

**Fig 4:**
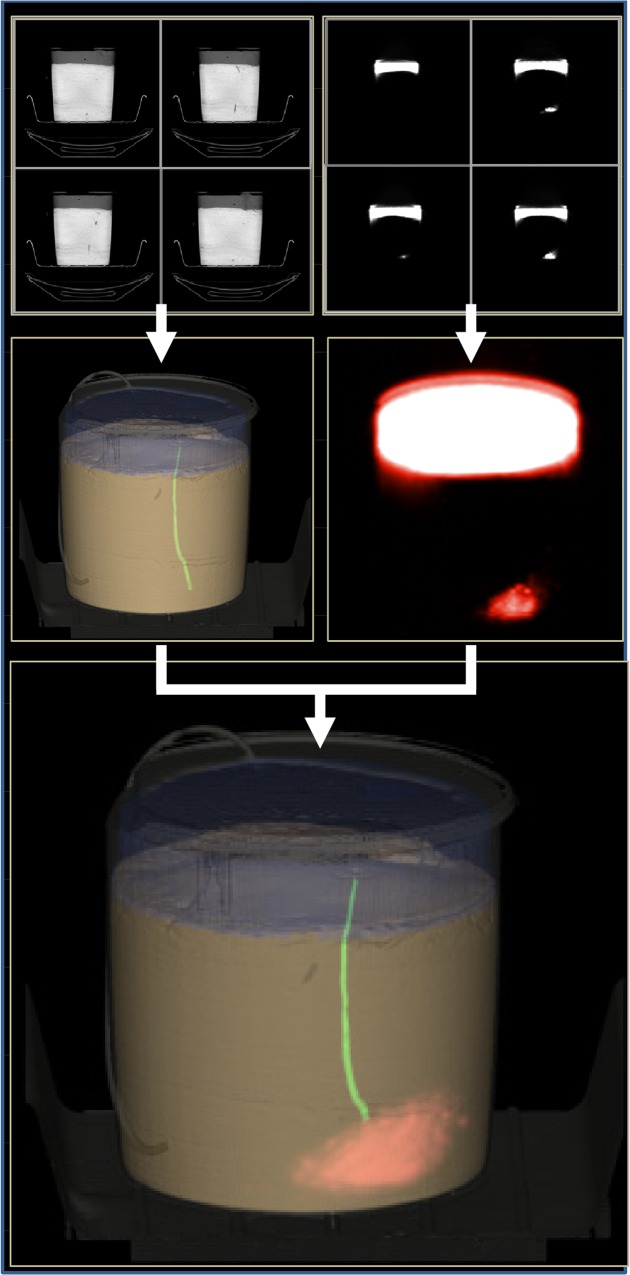
Construction of 3D images showing bioirrigating lugworm in sediment. A series of 2D cross-sections were assembled by computer to create full 3D images of the object (CT) and water injected with tracer (PET). Finally the co-registered 3D images are combined to form the resulting picture after 37 min. Artificial colors are used to highlights different components. The bucket was 25 cm in diameter.

The morphology of burrows was clearly visible on the CT images (Fig 4), but the lugworms were not identifiable because of too small difference in attenuation between the lugworm and water. Burrows were blind-ended and connected to the overlying water by one opening at the sediment surface. The architecture of 5 burrows ranged from a vertically (I-shape) or about a 45° angle (J-shape) descending from the burrow opening to more irregular and complex three-dimensional galleries. Overall, the average length of the burrows was 21 cm and the feeding pocket was located at an average depth of 14 cm. Larger worms had proportionally wider burrows (R^2^: 0.7, p = 0.055, Table 1).

**Table 1:** Lugworm body weight and burrow dimensions as measured from the CT images.

Name	Body weight (g)	Mean diameter (mm)	Total length (cm)	Feeding pocket depth (cm)
**Worm 1**	0.67	2.0 ± 0.3	28.5	15.8
**Worm 2**	1.00	2.2 ± 0.6	19.3	19.0
**Worm 3**	1.84	3.0 ± 0.6	20.0	9.6
**Worm 4**	2.62	2.7 ± 0.7	17.4	14.1
**Worm 5**	2.81	3.4 ± 0.7	21.1	11.5

The standard deviation is reported for the burrow diameter (n = 10–30).

### 4D visualization of bioirrigation

**Fig 5:**
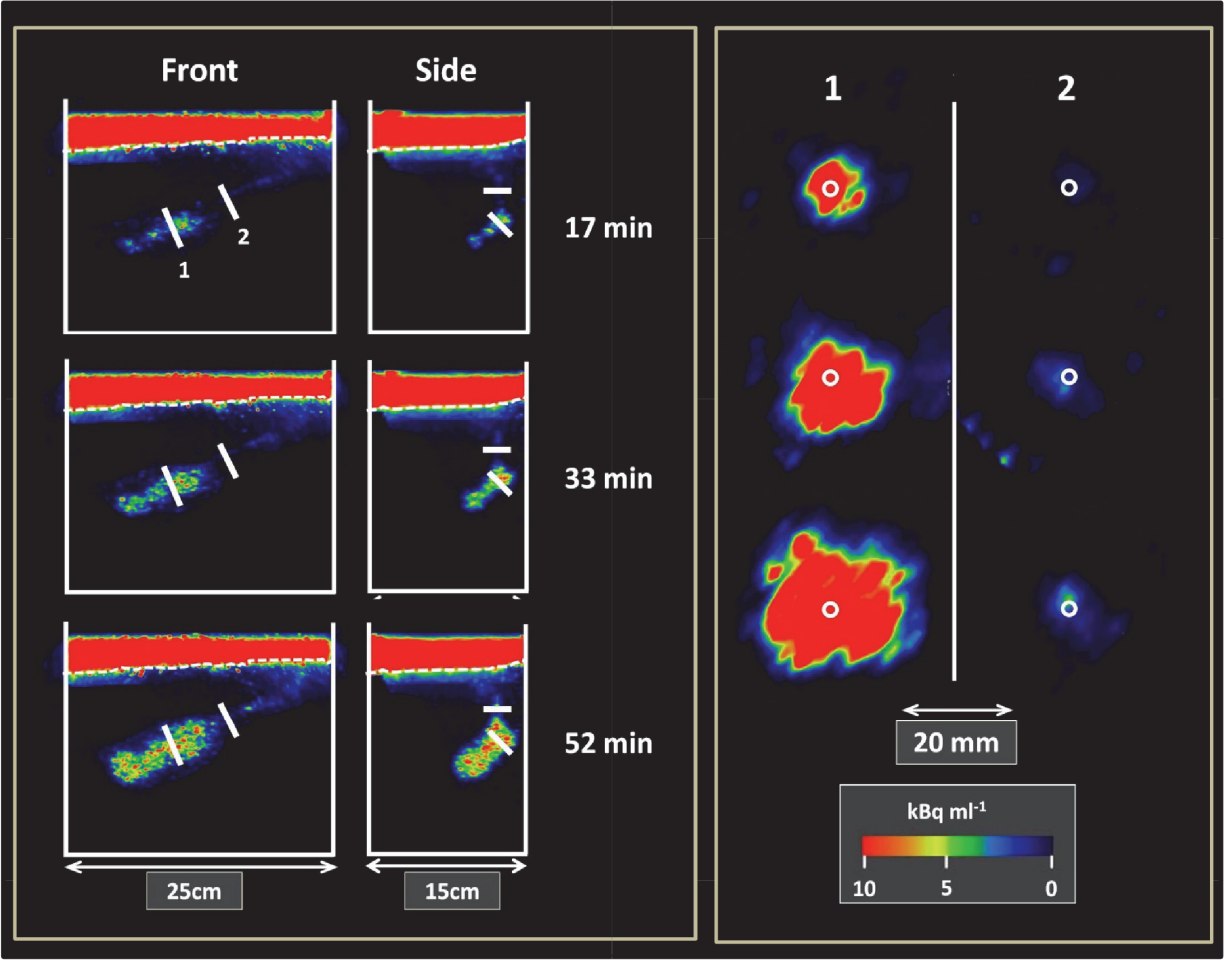
Images of the temporal change in radial bioirrigation. Left panel: Front and side view of a sediment compartment at different times showing the two cross sections (1: near the feeding pocket; 2: along the burrow shaft) of the burrow. The dashed lines indicate the sediment water interface. Right panel: Development of the bioirrigated area at cross section 1 and 2 after 17, 33 and 52 min (from top to bottom). The circles indicate the burrow circumference. Note the different scales in the two panels.

Three out of the 5 worms were actively pumping during the 1 hour scanning time. The combined 3D pictures obtained from PET and CT provided dynamic and detailed information of the spatial distribution of ^18^F relative to the burrow position as outlined by the CT images (Figs 4 and 5; S1 and S2 Movies). Water transport within the sediment radiated from the feeding pocket of the burrow, but with heterogeneous size and shape of the generated plume within the sediment. In some cases, the plume was localized only around the feeding pocket, and in other cases it was also evident 1–2 cm upward along the burrow shaft (Fig 5).

### Ventilation pattern

**Fig 6:**
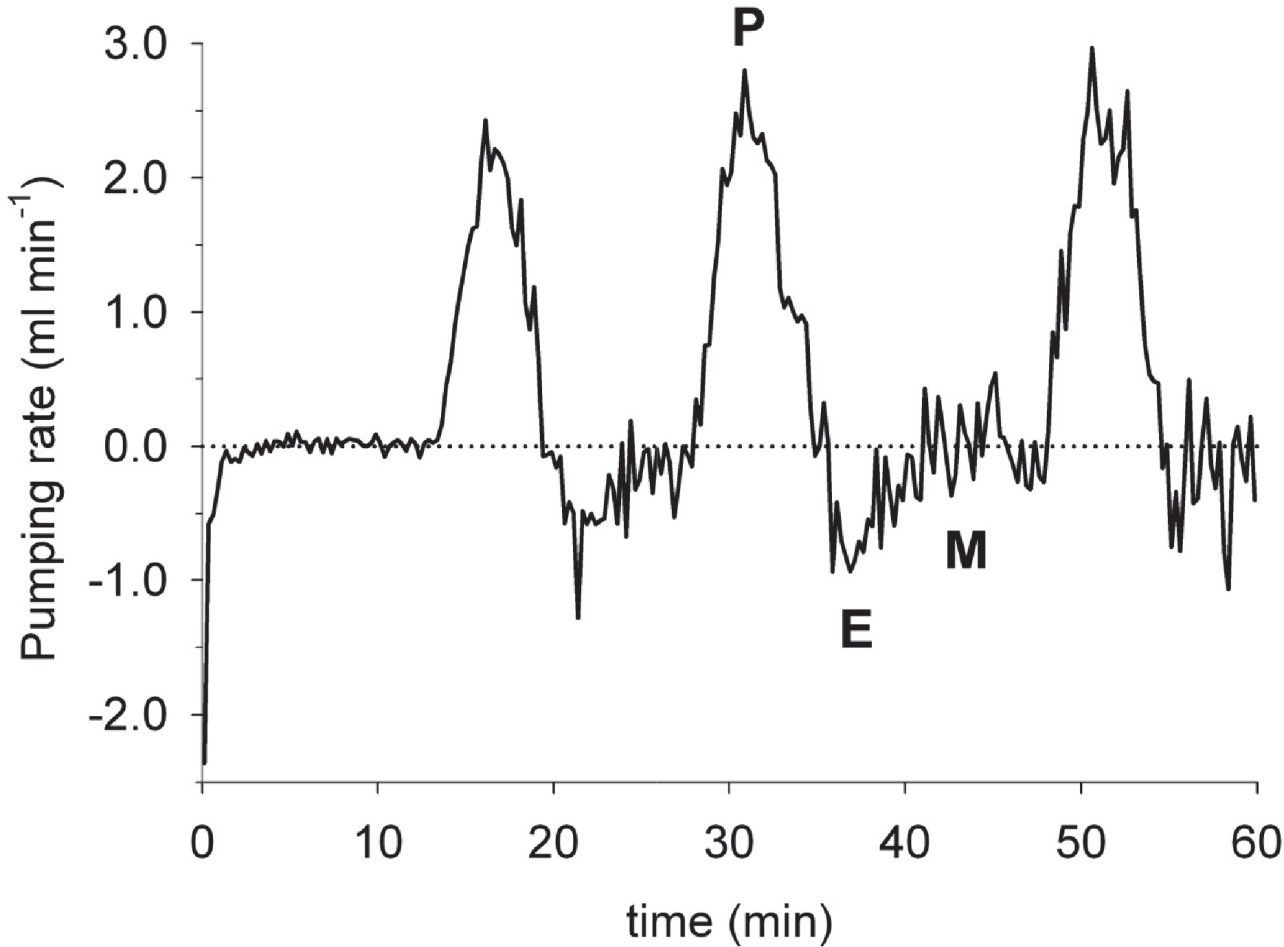
Ventilation pattern of lugworm number 3 (see Table 1) with pumping (P), reverse ejection (E) and maintenance (M) periods. The dotted line represents the zero baseline. The initial resting phase is probably due to a stress response after bucket movement.

The high frequency of our recordings enabled a detailed description of lugworm ventilation patterns (Figs 5 and 6; S3 Movie). Distinct periods of downward ventilation typically alternated with reverse ejections and periods of quiescence. The duration of these activity phases varied among lugworms, but the ventilation time appeared inversely related to ventilation intensity (Table 2; ρ_spearman_ = 0.43). On average, the animals ventilated during 33 to 80% of the elapsed time at a rate of 0.17 to 1.33 ml min^-1^. Overall the total amount of water pumped into the sediment by the lugworms ranged from 8 to 26 ml h^-1^ and was not clearly related to worm body mass (Table 2). Based on the obtained rates and burrow dimensions it was estimated that water in the burrow was renewed 1 to 4 times per minute during active ventilation.

**Table 2:** Lugworm ventilation parameters based on the combined results from PET and CT imaging.

Name	Body weight (g)	Ventilation rate per period (ml/min)	Average ventilation rate (ml/min)	Flow velocity (cm/min*)	Ventilation time per period (min)	Total ventilation time (min)	Total water ventilated (ml)
**Worm 2**	1.00	0.17	0.17	14.0	48	48	8.2
**Worm 3**	1.84	1.2	1.33	74	5.5	19.8	26.5
		1.31			7.5		
		1.48			6.8		
**Worm 4**	2.62	0.15	0.35	15.6	12.7	36.7	12.7
		0.45			18.7		
		0.45			5.2**		

* the average burrow section was calculated from Table 1.

** the experiment ended before the ventilation period was finished.

The duration of the experiments was about 1 h.

## Discussion

### Validation of the method

The CT approach used in this study provides for the first time a 3D image of lugworm burrows. The results confirm that *Arenicola marina* live in blind-ended burrows, but with a more complex morphology than the simple J-shape traditionally described for this species [13]. Image analysis provided additional information on the dimensions of lugworm burrows (length, diameter), confirming that CT is not only an exciting visualizing approach, but also an excellent measuring tool. The burrow dimensions observed in our study were in the low range of those measured directly by others probably owing to the relatively small size of the lugworms in our experiment [27].

CT has been increasingly used in marine science within the last decades to provide 3D visualization and measurement of biogenic structures created by various macroinvertebrates in muddy sediment [25,26]. Measurements of biogenic structures using CT image analyses currently represent the only non-destructive method that can provide the required high resolution (<1 mm) of burrow structures [25,28]. Perez et al. [11] (1999) noted that it is difficult to obtain valid CT images across a 15 cm wide core of sand. This problem was also to some extent apparent in our study, but image quality remained sufficient to visualize burrows through a 25 cm sand column.

The combination of PET and CT revealed for the first time the 3D and dynamic nature of bioirrigation induced by *A*. *marina*, which otherwise may require several invasive techniques [5,30]. The pumping rates obtained during active ventilation periods are in the low range of the rate measured by others—likely due to the small size of the animals used in our experiment [29]. PET images also revealed that the behavior of lugworms consisted of a succession of ventilation (5–20 min) and reverse ejection periods (1–4 min) that is typical for lugworms ([31–34]). Upward ejection of water in the tail shaft occurs either directly as backward pumping or through backward movement when the animal defecates at the surface. The quiescent periods (4–8 min) observed after reverse ejection is likely the result of feeding and burrow maintenance [32]. These results confirm and extend the application of the PET techniques described by Roskosch et al. [12] in muddy sediment.

### Potential applications

The large applicability of PET/CT scan opens a new window of opportunities for the understanding of bioturbation by benthic macrofauna and its ecological implications. PET images provide valuable information on the irrigation pattern and capacity of hidden animals that lives in blind-end burrows such as the lugworm, *A*. *marina*, but it is also applicable for animals living in open-ended burrows as shown by Roskosch et al. [12]. The ventilation of the blind-ended burrow of the lugworm was estimated based on the tracer transport and accumulation in the sediment. However, a technique based on ^18^F cannot be applied for open-ended burrows because most of the tracer passes rapidly through the burrow without significant decay [12]. We suggest that this problem can be solved using short-lived radionuclides such as ^15^O labeled water (t_½_ < 2 min) that are supplied continuously to the overlying water until steady state is reached. The radionuclide decay should then be equal the transport of water into the burrow, and the volume of water pumped at any given time can be calculated.

PET/CT scan also constitutes a valuable tool for experimental validation of bioirrigation modeling [8,29,32,35]. One implication of the results obtained here is that non-localized bioirrigation should be considered in future modeling studies where homogeneous bioirrigation often constitutes one of the basic assumptions. Enhanced flow might occur through cracks or inhomogeneities in the sediment surrounding burrows [36]. Such fine scale heterogeneity can be precisely elucidated by the images obtained from CT scan [37].

Sediment reworking can potentially be traced by PET using particle bound radioisotopes [7,38]. However, we recommend using CT instead because of its greater spatial resolution. 3D quantification and visualization of sediment reworking has been achieved following aluminum oxide particle transport [26]. But, we hypothesized that the complementary capacities of PET/CT provide a complete 4D assessment of bioturbation [1], where sediment reworking can be visualized/quantified by CT and ventilation and irrigation by PET.

**Fig 7:**
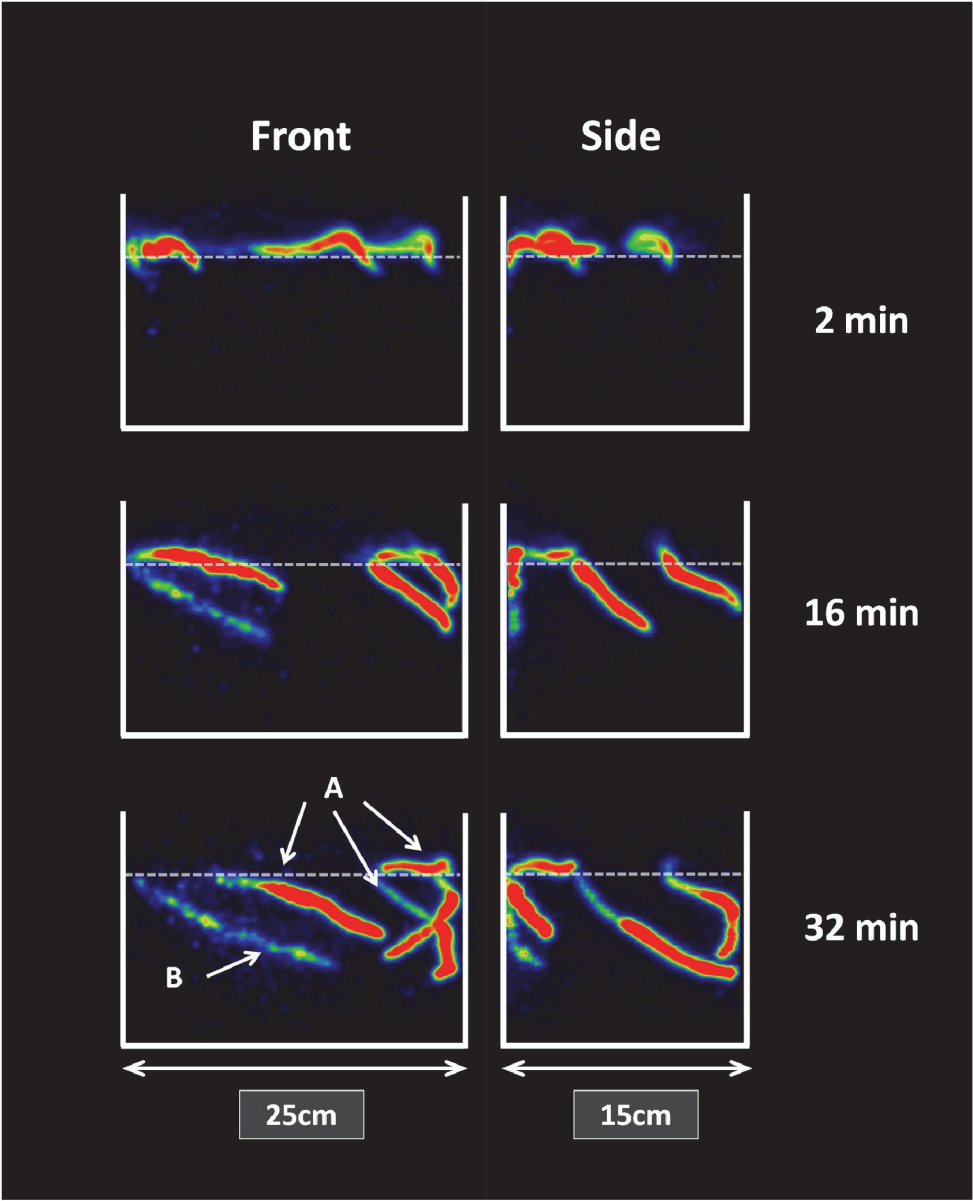
3D visualization of lugworm, *Arenicola marina*, movements within the sediment. Front and side images of ^18^F injected lugworm, *Arenicola marina*. The worms were injected with (A) 150 μl and (B) 50 μl of ^18^F solution and placed at the surface. PET pictures acquired every minute show the burial behavior and speed of the lugworms. The dashed lines indicate the sediment water interface. Front (25 cm) x Side (15 cm).

Furthermore, Injection of radionuclides directly into burrowing animals opens the possibilities to trace their behavior when hidden inside the substratum. PET can record animal position and movements within sediments at high temporal frequency. Intra- or interspecific interactions among animals can be studied in great detail by injecting different tracers or concentrations into different individuals or species (Fig 7; S4 Movie). The stress effect of injection and toxicity of tracer on the animals should be taken into account, but a pilot study revealed that there is no behavioral and survival effect from injection of 50–150 μl of Na^18^F (80 MBq diluted in 1 ml) into the coelom of lugworms. This technique could substitute the use of transparent and artificial substratum, like gelatin, to observe the behavior of burrow-dwelling animals [36].

Other radiographic solutions have been used to visualize animal position within the sediment. Charbonneau et al. [39] were able to visualize the location of mayfly larvae (*Hexagenia* spp.) on X-ray images by placing a mixture of glue and metal (Mo_2_C) on the animals. This rather invasive approach could be applied with a CT and provide 3D visualization. Dufour et al. [25] instead suggested the direct use of CT to observe the position of animals in the sediment. In this way, Mermillod-Blondin [37] could actually distinguish between shells of *Mya arenaria* and *Macoma balthica*, but soft tissue animals, like lugworms, are not visible with CT.

Another promising tool in the 3D exploration of biological processes in sediments is MRT (magnetic resonance tomography) or also called MRI (magnetic resonance imaging). Although it is based on completely different principles than CT [40], it can provide structural information at similar scale as CT. MRI is better than CT at distinguishing between soft tissues and Kleinhans et al. [41] suggested that it should be possible to distinguish between animals and the water inside burrows. This postulate remains to be tested, but Bauman et al. [42] used MRI to measure advection and diffusion processes in sediment without any tracers. A more advanced PET/MRI combination is advantageous compared with PET/CT because MRI and PET images can be recorded simultaneously without moving the object. Application of MRI or PET/MRI has to our knowledge not been proven for marine bioturbated sediment.

The major drawback of the tomographic techniques is the high economic costs. Only few institutions have the capacity to acquire and maintain such expensive and advanced equipment. The cost of one clinical examination using PET/CT scan in Denmark is currently about 1500 €. This means tomographic-based studies will only be possible if ecologists collaborate with nuclear medicine specialists or radiologists [11]. Since its first use in marine ecology by Perez et al. [11] in 1999, tomography based-studies have tackled challenging issues that could not be elucidated otherwise.

## Supporting Information

S1 File Sediment carbonate, iron and clay content. (DOCX)

S1 Movie 360-rotation of the combined PET-CT pictures of the sediment bucket 1 hour after ^18^F injection in the overlying water. The plastic of the bucket, the sand and burrow were artificially colored based on their different density measured by the CT images. The ^18^F-Fluoride rich water is highlighted in red based on the PET images. The bucket diameter was approx. 25cm.
(WMV)

S2 Movie Combined 3D-PET-CT pictures 1 hour after ^18^F injection in the overlying water. The sand and burrow were artificially colored in brown and green based on CT images and ^18^F-Fluoride rich water was colored red (porewater) and blue (overlying water). The bucket diameter was approx.25cm.
(WMV)

S3 Movie Time-lapse projected images (front and side views) of the temporal change in radial bioirrigation. The dashed lines indicate the sediment water interface identified by the CT images.
(WMV)

S4 Movie Time-lapse projected images (front and side views) of the 18F injected lugworm movement in the sediment. The dashed lines indicate the sediment water interface identified by the CT images.
(WMV)
