# Supplementary material for: Seeing the Unseen—Bioturbation in 4D: Tracing Bioirrigation in Marine Sediment Using Positron Emission Tomography and Computed Tomography
Source: PLoS One. 2015 Apr 2;10(4):e0122201. doi: 10.1371/journal.pone.0122201 (PMC4383581; doi:10.1371/journal.pone.0122201)
Supplement: S1 File — (DOCX) [file pone.0122201.s001.docx]

The sediment physical and chemical characteristics were measured in parallel of a survey of the benthic fauna Odense Estuary presented in Kristensen et al. [1]. The sediment data referred to in the submitted MS are not published to this date.

**Methods:**

Sediment was collected in 5cm-platic cores. Upon arrival in the laboratory the cores were sectioned in several layers representing 0-1cm, 1-2cm and 2-6cm [1]. The data presented below are depth integrated for the top 6 cm.

**Mud and clay:** Grain size was measured from about 20 g of wet sediment that was passed through a Wentworth series of sieves of decreasing size. The mud and clay fraction represent the dry weight proportion of the sediment with grain size < 63 µm[2].

**Iron:** Iron content (TFe) of the ashes were measured by extraction in 1M HCl (105°C; 30min). TFe of the sediment was measured by spectrophotometry at 562nm [3]. The sample was added to a solution of ferrozine that form a pink complex with ferrous iron liberated by acidification of the ashes. TFe was ontained in μg/g_ash_ combusted and converted into μg/gDW based on organic matter contents measurements.

**Carbonate:** Carbonate content was estimated by decarboxylation, where about 0.5g of combusted sediment was transferred into Pyrex tubes containing 5ml of 1M HCl. The tubes were heated for 30 minutes into boiling water and checked for no persistent CO2 liberation. The supernatant was collected for iron and phosphorus measurements. To avoid the hydroscopic effect of the hydrochloric acid, the sediment was rinsed with distilled water and centrifuged three times (3000rpm; 5min). Finally, the residual was dried and the carbonate content estimated as the weight loss and was ontained in μg/g_ash_ combusted and converted into μg/gDW based on organic matter contents measurements.

**Results:**

| Station | N | E | OM(0-6) | TFe µg/g DW | %mud_0_63 | Carbonate |
| --- | --- | --- | --- | --- | --- | --- |
| OF127 | 55.4914 | 10.6183 | 0.50 | 309 | 2.0 | 3.5 |

Reference List

1. Kristensen E, Delefosse M, Quintana CO, Banta GT, Petersen HC, Jørgensen B (2013) Distribution pattern of benthic invertebrates in Danish estuaries; the use of Taylor's power law as a species-specific indicator of dispersion and behaviour. J Sea Res 77: 70-78.

2. Blott SJ, Pye K (2001) GRADISTAT: A grain size distribution and statistics package for the analysis of unconsolidated sediments. Earth Surface Processes and Landforms 26: 1237-1248.

3. Stookey LL (1970) Ferrozine - A new spectrophotometric reagent for iron. Anal Chem 42: 779-&.
